# Gender Differences in Liver Steatosis and Fibrosis in Overweight and Obese Patients with Metabolic Dysfunction-Associated Steatotic Liver Disease before and after 8 Weeks of Very Low-Calorie Ketogenic Diet

**DOI:** 10.3390/nu16101408

**Published:** 2024-05-08

**Authors:** Roberta Rinaldi, Sara De Nucci, Rossella Donghia, Rosanna Donvito, Nicole Cerabino, Martina Di Chito, Alice Penza, Francesco Pio Mongelli, Endrit Shahini, Marianna Zappimbulso, Pasqua Letizia Pesole, Sergio Coletta, Vincenzo Triggiani, Raffaele Cozzolongo, Gianluigi Giannelli, Giovanni De Pergola

**Affiliations:** 1 Center of Nutrition for the Research and the Care of Obesity and Metabolic Diseases, National Institute of Gastroenterology IRCCS “Saverio de Bellis”, Castellana Grotte, 70013 Bari, Italy; roberta.rinaldi@irccsdebellis.it (R.R.); sara.denucci@irccsdebellis.it (S.D.N.); rosanna.donvito@irccsdebellis.it (R.D.); nicole.cerabino@irccsdebellis.it (N.C.); dichitomartina@gmail.com (M.D.C.); alicepenza@gmail.com (A.P.); francescomongio@gmail.com (F.P.M.); 2 Laboratory of Epidemiology and Statistics, National Institute of Gastroenterology “Saverio de Bellis”, IRCCS Hospital, Castellana Grotte, 70013 Bari, Italy; rossella.donghia@irccsdebellis.it; 3 Department of Gastroenterology, National Institute of Gastroenterology “Saverio de Bellis”, IRCCS Hospital, Castellana Grotte, 70013 Bari, Italy; endrit.shahini@irccsdebellis.it (E.S.); marianna.zappimbulso@irccsdebellis.it (M.Z.); raffaele.cozzolongo@irccsdebellis.it (R.C.); 4 Core Facility Biobank, National Institute of Gastroenterology “Saverio de Bellis”, IRCCS Hospital, Castellana Grotte, 70013 Bari, Italy; letizia.pesole@irccsdebellis.it (P.L.P.); sergio.coletta@irccsdebellis.it (S.C.); 5 Interdisciplinary Department of Medicine, School of Medicine, University of Bari “Aldo Moro”, Piazza Giulio Cesare 11, 70124 Bari, Italy; vincenzo.triggiani@uniba.it; 6 Scientific Direction, National Institute of Gastroenterology “Saverio de Bellis”, IRCCS Hospital, Castellana Grotte, 70013 Bari, Italy; gianluigi.giannelli@irccsdebellis.it

**Keywords:** non-alcoholic fatty liver disease (NAFLD), metabolic dysfunction-associated steatotic liver disease (MASLD), very low-calorie ketogenic diet (VLCKD), obesity, transient elastography (FibroScan), gender

## Abstract

Obesity and metabolic syndrome are linked to steatotic liver disease (SLD), the most common form of chronic liver disease. Lifestyle modifications and dieting are strategies that can prevent metabolic dysfunction-associated steatotic liver disease (MASLD). The very low-calorie ketogenic diet (VLCKD) is a helpful treatment for MASLD and has been recommended for people affected by obesity; we evaluated the effect of gender on steatosis and fibrosis in a cohort of 112 overweight or obese patients undergoing an eight-week treatment with a VLCKD. Differences between the genders in terms of anthropometric measures, body composition, and metabolic indicators were examined before, during, and after the nutritional intervention. At baseline, there were significant differences between men and women in terms of anthropometric parameters, blood pressure, Homeostatic Model Assessment for Insulin Resistance (HOMA-IR), fasting insulin, hepatic markers, and lipid profile. Men had considerably higher levels of liver steatosis (measured by CAP) and liver stiffness (measured by E) under basal conditions than women. After the VLCKD, there were reductions in both genders of controlled attenuation parameter (CAP), body weight, body mass index (BMI), waist circumference, systolic and diastolic blood pressure, insulin resistance, fat mass (FM), free fat mass (FFM), and fasting blood glucose, insulin, glycated hemoglobin (HbA1c), triglycerides, total cholesterol, low-density lipoprotein (LDL) cholesterol, alanine transaminase (ALT), gamma-glutamyl transferase (γGT), and uric acid levels. Only in men, liver stiffness, aspartate aminotransferase (AST), creatinine, and C-reactive protein (CRP) levels significantly decreased. Moreover, men had significantly greater levels of liver steatosis: the male gender featured an increase of 23.96 points of the Fibroscan CAP. Men exhibited higher levels of steatosis and fibrosis than women, and these differences persist despite VLCKD. These gender-specific variations in steatosis and fibrosis levels could be caused by hormonal and metabolic factors, suggesting that different therapeutic strategies might be required depending on the gender.

## 1. Background

Over the past few decades, obesity has become a global public health concern [1]. The prevalence of obesity is increasing steadily in Italy, where the percentage of adult obese people has increased by almost 30% in recent years [2]. Moreover, overweight individuals may have an increased risk of developing comorbid conditions, such as metabolic syndrome, type 2 diabetes, and liver disease [3].

Many aspects of obesity, including body composition, lipid metabolism, endocrine function, and low-grade chronic inflammation, are impacted by gender differences [4]. Gender differences in obesity may be explained by socioeconomic and cultural status, comorbidities related to hormones, and metabolic issues [5]. Obesity-related complications typically affect men more frequently [6,7,8]. Overweight and obesity have different pathological characteristics between males and females. Therefore, weight loss strategies are likely to have different effects on male and female subjects [9]. These data have recently been confirmed in a review by Muscogiuri et al., for whom the prevalence of obesity among men is higher than in women. Anyway, women have a higher percentage of body fat content compared to men; in fact, gender appears to be an important factor in the manifestation of central (android) or peripheral (gynoid) obesity. Weight loss has similar but distinct effects: women have to face more psychological obstacles, while men have fewer comorbidity benefits [10].

The development of the metabolic syndrome and its pathophysiological effects, including insulin resistance, cardiovascular disease, and other non-metabolic obesity-related complications, are caused by a state of chronic low-grade inflammation induced by obesity [11]. Obesity and hepatic steatosis are both components of the metabolic syndrome and are closely related. Over 30% of people worldwide suffer from non-alcoholic fatty liver disease (NAFLD), and the incidence is as high as 70% in people with metabolic disorders [12,13]. Metabolic dysfunction-associated steatotic liver disease (MASLD) is the term currently used to describe the co-occurrence of these diseases, along with at least one of five cardiovascular risk factors indicative of the metabolic syndrome.

In western adults, these five criteria include the following: BMI ≥ 25 kg/m^2^ or WC > 94 cm in men and 80 cm in women; fasting serum glucose ≥ 5.6 mmol/L (100 mg/dL) or 2 h post-load glucose levels ≥ 7.8 mmol/L (≥140 mg/dL) or HbA1c ≥ 5.7% (39 mmol/L), or type 2 diabetes or treatment for type 2 diabetes; blood pressure ≥ 130/85 mmHg or specific antihypertensive drug treatment; plasma triglycerides ≥ 1.70 mmol/L (150 mg/dL) or lipid-lowering treatment; plasma HDL-cholesterol ≤ 1.0 mmol/L (40 mg/dL) in men and ≤1.3 mmol/L (50 mg/dL) in women or lipid-lowering treatment [13,14].

Moreover, increased liver stiffness measured by transient elastography was observed more frequently in men and was associated with metabolic syndrome features [15,16].

Men have a higher prevalence and severity of NAFLD than women during their reproductive years. Nonetheless, NAFLD affects women more frequently after menopause, indicating that estrogen may offer some protection [17,18]. In contrast to other studies, our prior work revealed that while males were more likely to have this disease within our cohort, females with obesity and steatosis showed worse clinical outcomes. Therefore, it would seem that the effects of steatosis and obesity are distinct, and the variations observed in that research are likely due to variations in hormonal or genetic profiles [19]. In addition, men are more likely to develop hepatocellular carcinoma and have worse outcomes [20].

A healthy diet and increased physical activity are two weight-loss-oriented lifestyle changes that can help prevent MASLD. These modifications enhance insulin sensitivity, lower systemic inflammation, encourage weight loss, and decrease body fat [21]. As previously shown, a concurrent decrease in white blood cells and platelets, low-grade inflammatory expression, liver steatosis, and liver fibrosis occurs in overweight and obese individuals receiving VLCKD treatment [22].

Very low-calorie ketogenic diets offer a substantial, quickly achieved, and tolerable reduction in body weight as a nutritional therapy for extreme obesity [23]. A very low-calorie intake (400–800 kcal/day) combined with a very low carbohydrate intake (<50 g/kg/day) characterizes VLCKDs. Promoting a shift in energy metabolism from carbohydrates to triglycerides and the production of ketone bodies is the goal of VLCKDs [24]. Compared to balanced low-calorie diets, this leads to weight loss more quickly [25]. Thus, MASLD patients have a very helpful option for reducing liver steatosis due to the quick mobilization of fat from adipose tissue and weight loss brought on by VLCKD [26]. The effects of VLCKD on NAFLD have already been recognized in subjects with insulin resistance, who benefit more than subjects without insulin resistance [27].

The main objective of this study is to examine the impact of gender on steatosis and fibrosis in a cohort of 112 overweight/obese adults who underwent eight weeks of VLCKD. Additionally, differences between men and women were analyzed in terms of anthropometric measurements, body composition, and metabolic biomarkers at T0 and after 8 weeks of VLCKD.

## 2. Materials and Methods

### 2.1. Study Design and Population

This 8-week real-life prospective study was conducted by our Centre of Nutrition for the Research and Care of Obesity and Metabolic Diseases of the National Institute of Gastroenterology at Saverio De Bellis Research Hospital (Castellana Grotte, Bari, Apulia, Italy).

Overweight or obese patients aged 18–65 years with a body mass index (BMI) of at least 25 kg/m^2^ [13] underwent close analysis of the medical history, an anthropometric assessment, physical examination, and laboratory tests. During the medical history collection, subjects were questioned about smoking habits, and their daily alcohol consumption was examined based on American and European recommendations, asking patients directly about the number of glasses of alcohol they drank per day: two glasses of alcohol per day for male patients and one glass of alcohol per day for female patients [28,29], defining a threshold of 30 g/day for men and 20 g/day for women.

Exclusion criteria included a list of contraindications for a VLCKD [30,31,32]: hypersensitivity to components contained in meal replacement products, type 1 diabetes mellitus, a history of cerebrovascular and cardiac diseases, respiratory insufficiency, severe GI diseases (i.e., inflammatory bowel disease, autoimmune diseases, cancer), chronic kidney disease characterized by an estimated glomerular filtration rate < 60, psychiatric issues, or pregnancy and lactation. Additional exclusion criteria were eating disorders and other serious mental illnesses, liver failure, substance abuse, frail elderly patients, active/severe infections, and rare disorders such as porphyria or deficiency of carnitine or carnitine–palmitoyl transferase or carnitine–acylcarnitine translocase or pyruvate carboxylase, and disorders of mitochondrial fatty acid oxidation. Patients who consumed more alcohol than recommended were also excluded.

The local Medical Ethics Committee approved this study’s protocol (Prot. n. 170/CE De Bellis); 111 people took part in this study, which was conducted in compliance with the 1964 Helsinki Declaration. Prior to participating in this study, each subject gave written informed consent. NCT05477212 is this study’s ClinicalTrials.gov identifier. Patients were enrolled from July 2022 to December 2023. Two check-ups were conducted: at baseline (T0) and eight weeks after the VLCKD (T1). Anthropometric measurements, fasting blood sample data, and results from instrumental testing (BIA and Fibroscan) were gathered at T0 and T1. The method of investigation has been discussed in previous papers [33].

### 2.2. Diet Protocol

The VLCKD protocol applied to the present study has been reported in a few of our earlier publications [22,33,34]. The first two steps were determined in accordance with the description published by Bruci et al. [35]. According to the two-step protocol technique from New Penta, Cuneo, Italy, each participant received the VLCKD, a multi-step dietary model with meal replacements for the management of obesity and related metabolic disorders. The ketogenic nutritional therapy (KeNuT) plan [31] states that the VLCKD is a very low-calorie (650–800 kcal/day), low-fat (20 g daily), and low-carbohydrate (<30 g daily from vegetables) diet; the main components of the VLCKD initial phase are based on olive oil. Research has shown that a set range of 1–1.4 g of protein per kg of ideal body weight per day can maintain lean mass and satisfy the body’s minimum daily demand. Participants were asked to consume less than eight hundred calories per day and drink at least two liters of water daily. Micronutrient-enriched supplements were given over the course of the dietary treatment since the diet was unbalanced and lacked fresh fruit [32]. Patients consumed high biological-value protein replacement meals four or five times a day during phase 1. To guarantee the appropriate quantity of fiber, only vegetables with a low sugar and low glycemic index were consumed. Herbs and spices, lemon, and extra virgin olive oil (two teaspoons daily) were permitted throughout the active phase. In Phase 2, the calorie consumption increased somewhat but remained similar to the previous step. In fact, the purpose was to retrain the patients to eat fresh protein, progressively weaning them off the replacement meals in order to sustain nutritional ketosis. A natural protein meal was deliberately reintroduced. There were two distinct sub-phases during this stage. In the first, we reminded the patients that dairy products were still prohibited and offered a single meal replacement (lunch or dinner) consisting of fresh protein from meat, fish, and eggs; during the second sub-phase, both meals were replaced with fresh protein.

### 2.3. Anthropometric Parameters, Bioelectrical Impedance Analysis (BIA), and Biochemistry

Body weight and height were assessed in fasting volunteers, who were barefoot, dressed in light clothing, and had an empty bladder in order to calculate the BMI (kg/m^2^). The same stadiometer and calibrated scale were then used to measure each subject. To measure their waist circumference (WC), patients undressed and stood with their feet close together. The circumferential point was located in the iliac crest and the lower rib margin. An OMRON M6 automated blood pressure monitor was used to measure the diastolic (DBP) and systolic (SBP) pressures three times with the subject at rest in a sitting position. At baseline, three weeks into the VLCKD treatment, and then eight weeks later, every anthropometric measure was collected.

Moreover, a trained dietitian performed the bioelectrical impedance analysis (BIA) applying standard procedures: before the examination, participants were asked not to eat or drink anything for 12 h and not to exercise for 24 h. During the examination, they were lying supine with their legs apart [36,37], and the electrodes were attached to the cleansed areas. BIA was performed using a single-frequency bioimpedance analyzer (BIA-101 analyzer, 50-kHz frequency; Akern Bioresearch, 168 Florence, Italy), and Akern software (BODYGRAM^®^ PLUS) was used to calculate body composition parameters.

Blood samples were collected between 8:00 and 9:00 a.m., following an overnight fast: the biochemical conditions were measured both before and after the VLCKD, using standard laboratory methods. Using the COBAS 8000 autoanalyzer (ROCHE 182 Diagnostic SPA, Monza, Italy), the concentrations of insulin, triglycerides, total cholesterol, LDL cholesterol, HDL cholesterol, AST, ALT, γGT, and 25-OH vitamin D were determined together with fasting blood glucose. HbA1c was measured using the Capillarys 3 OCTA automatic capillary electrophoresis system (Sebia Italia S.r.l., Bagno a Ripoli, Firenze, Italy). The Homeostasis Model Assessment–Insulin Resistance (HOMA-IR) tool was used to quantify insulin resistance [38].

### 2.4. Fibroscan and NAFLD Assessment

Studies conducted on obese people indicate that Fibroscan provides a quick and painless assessment of liver conditions, eliminating the need to perform a liver biopsy, an invasive procedure [39], and are equally accurate in diagnosing the existence of hepatic steatosis: liver fibrosis is seen when liver stiffness values are greater than 8 kPA, and mild SLD is visible when CAP is greater than 275 dBm [40]. CAP (Echosens, Paris, France) was used to assess the amount of hepatic fat-induced ultrasonic attenuation at the standard frequency of 3.5 MHz [41].

### 2.5. Statistical Analysis

Patients’ characteristics are reported as mean and standard deviation (M ± SD) for continuous variables and as frequency and percentages (%) for categorical variables. Wilcoxon matched-pairs signed-rank test for continuous variables was applied to evaluate variations in pre/post ketogenic diets, while for categorical variables, the McNemar-Bowker test was applied.

In addition, to test the association between independent groups (Male Before Diet vs. Female Before Diet or Male After Diet vs. Female After Diet), the Wilcoxon rank-sum (Mann–Whitney) test was used for continuous and the Chi-Square test for categorical variables.

A linear regression model of the Fibroscan CAP on gender (male vs. female) was used to evaluate the association between liver parameters and gender, and the covariates (Age, Smoking, BMI, and HOMA) were used to adjust the model.

To test the null hypothesis of non-association, the two-tailed probability level was set at 0.05. The analyses were conducted with StataCorp. 2023. Stata Statistical Software: Release 18. StataCorp LLC.: College Station, TX, USA.

## 3. Results

Cohort and liver characteristics stratified by gender are shown in Table 1 and Table 2, respectively. The female gender was predominant (66.96% vs. 33.05%), and males were older (42.46 ± 11.69 vs. 41.48 ± 13.15), although the difference was not significant.

In basal conditions, liver steatosis (measured by CAP), liver stiffness (measured by E), body weight, BMI, waist circumference, FFM, systolic and diastolic blood pressures, insulin resistance (expressed as HOMA), fasting insulin, triglycerides, AST, ALT, γGT, ferritin, uric acid, creatinine levels, and percent of smokers were significantly higher in men than women, whereas HDL-cholesterol concentrations were higher in women.

After the VLCKD, CAP, body weight, BMI, waist circumference, systolic and diastolic blood pressure, insulin resistance, FM, FFM, fasting blood glucose, insulin, HbA1c, triglycerides, total cholesterol, LDL-cholesterol, ALT, γGT, and uric acid were lower in both genders. Liver stiffness, AST, creatinine, and CRP levels were only significantly reduced in men.

After the VLCKD, liver steatosis, body weight, waist circumference, FFM, diastolic blood pressure, fasting triglycerides, AST, ALT, γGT, ferritin, uric acid, creatinine, and HDL-cholesterol levels were significantly higher in men, whereas CRP levels were higher in women.

An association of CAP with gender was found (Table 3). The male gender features an increase by 23.96 points of the Fibroscan CAP (β = 23.96, *p* = 0.019, 4.00 to 43.91 95% C.I.) at baseline.

## 4. Discussion

The aim of this study, performed in a group of patients from Southern Italy characterized by steatotic liver disease and obesity, was to explore gender-based differences in liver steatosis and fibrosis, hormones and metabolic blood variables, and anthropometric parameters before and after 8 weeks of VLCKD.

Our data show that in basal conditions, the level of steatosis and fibrosis was higher in men. Men also have higher liver-related hematochemical parameters, in line with their higher degree of steatosis: in fact, AST, ALT, and γGT levels were higher in men than women. Therefore, it is clear that men are more prone than women to developing steatosis and fibrosis. Accordingly, we could suggest that overweight and obese men need more specific attention in the prevention and care of MASLD.

Obesity is known to be more prevalent in women than in men. Compared to men, women are more likely to have an obesity diagnosis and seek out and receive any treatment for their condition [42].

At the same time, men had a higher BMI and central fat (measured by waist circumference) than women.

Systolic and diastolic blood pressure, insulin resistance (measured by HOMA), insulin, triglycerides, uric acid, ferritin, and creatinine blood levels were also higher in men, while HDL-cholesterol levels were lower, thus contributing to a higher cardiovascular risk of patients with liver steatosis and fibrosis. Some or all of these parameters may contribute to higher steatosis and/or fibrosis in men. Alternatively, it may well be that gender itself and/or sex hormones may be responsible for differences in liver steatosis and fibrosis.

After 8 weeks of VLCKD, the CAP was lower in both genders, while liver stiffness was significantly reduced only in men; however, the CAP was still higher in men than women, suggesting that changes in the BMI and HOMA index do not change the unfavorable influence of male gender on liver steatosis. In contrast to hepatic steatosis, liver stiffness was not significantly different between men and women after the VLCKD, suggesting that the reduction in liver fibrosis is greater in men than in women and that a VLCKD is more favorable in men than in women against liver fibrosis.

This finding is in line with a recent study showing that the fibrosis score could predict liver status in males but not in females [43]. Consistently, ALT and γGT were also improved in both genders, while AST decreased only in men.

It is possible that the anti-inflammatory characteristics of the VLCKD itself played a role in the decreased steatosis. Indeed, a meta-analysis found that inflammatory responses, and specifically cytokine expression (IL-2 and IL-6), differed according to gender [44]. The only inflammatory parameter evaluated in our study was CRP, but we found only a trend to a reduction after the VLCKD, with no significant effect of gender. Hyperferritinemia could be used as a marker of hepatic inflammation: in our results, both genders showed a statistically significant change after the VLCKD, with an increase in females and a decrease in males, indicating an apparent advantage for men.

After the VLCKD, body weight, BMI, and waist circumference were also lower in both genders. Men are more inclined than women to accumulate adipose tissue around the abdomen, while women typically do so around the hips and thighs [42]. As shown in previous studies [45], men had higher WC and FFM than women before and after VLCKD, and, accordingly, men had higher mean creatinine levels because of their greater muscle mass. This may be related to hormonal differences between the sexes. In fact, it is known that testosterone in men increases muscle mass by increasing muscle protein synthesis [46]. In contrast, even though both groups had a decreased fat mass, we did not find any difference in fat mass between the genders at baseline or following the VLCKD.

Lipid metabolism is different in the two sexes; in fact, women’s lipid profiles fluctuate more than men’s because of the complex hormonal changes that occur throughout their lives, particularly those associated with menopause and pregnancy. Human chorionic gonadotropin hormone, beta-estradiol, insulin, and progesterone all contribute to these alterations [47,48]. In our results, there was a statistically significant difference when comparing the two sexes in the increase in HDL fraction and the decrease in TG concentration; LDL and total cholesterol both dropped as a result of the rise in HDL.

Regarding the prevalence of hypertension, it increases in proportion to age in women, even though men are more likely to have it overall. This is because women’s blood pressure begins to rise sharply in their third decade of life [49]. Blood pressure is a sexually dimorphic trait, and we found that women tend to have significantly lower diastolic blood pressure than men, whereas systolic blood pressure was not different according to gender, suggesting that SBP decreases after a VLCKD more in men than in women.

In both sexes, weight loss and improved lipid balance achieved by VLCKD reduced pressure levels.

In regard to uric acid, it increased significantly in women after the VLCKD but not in men, indicating a further apparent advantage for men.

In terms of glycemic and insulinemic balance, women are more insulin-sensitive than men, but this metabolic benefit gradually diminishes after menopause or when insulin resistance develops into hyperglycemia and diabetes [50].

Insulin resistance is associated with a de novo increase in hepatic lipogenesis, which adds to the buildup of triglycerides [51]: after VLCKD, patients had decreased circulating insulin concentrations, endogenous glucose production, and hepatic insulin resistance, which led to increased liver triglyceride hydrolysis during the intrahepatic production of ketones [52]. Insulin levels and HOMA were not different between men and women after VLCKD, thus apparently excluding a significant effect of gender on insulin levels and insulin resistance.

### Strengths and Limitations

The data analysis for this study was conducted on a group of patients from Southern Italy with similar profiles, and in order to prevent any potential therapeutic interactions, this study only included participants who were not on any supplements or medication regimens. This gives this study methodological legitimacy. FibroScan, the only method approved by recommendations for assessing liver steatosis when biopsy is not possible, was used to estimate liver parameters, as it is commonly employed in suspected cases of hepatic steatosis.

There are various limits that must be understood. It would be desirable to divide the sample into different age groups and simultaneously evaluate hormonal and genetic factors in order to assess the changes they induce. In addition, psychological and lifestyle-related aspects should also be considered.

## 5. Conclusions

This study, performed in patients who were overweight and obese, shows that men present a higher level of steatosis and fibrosis compared to women and that higher levels of steatosis in men are maintained after a VLCKD. These gender differences in the level of steatosis and fibrosis might be due to hormonal and metabolic parameters and suggest that a different clinical approach may need to be proposed according to gender.

## Figures and Tables

**Table 1:** Epidemiological and clinical characteristics of patients undergoing the ketogenic diet, stratified by gender (*n* = 112).

Parameters *	Gender			Gender			*p* ^†^	*p* ^ѱ^
	F (*n* = 75)			M (*n* = 37)				
	Before	After	*p* ^^^	Before	After	*p* ^^^		
Age (yrs)	41.48 ± 13.15	--	--	42.46 ± 11.69	--		0.85	--
Weight (Kg)	92.53 ± 15.70	84.38 ± 14.04	<0.0001	110.66 ± 22.99	100.41 ± 21.38	<0.0001	<0.0001	<0.0001
BMI (Kg/m^2^)	35.28 ± 5.52	32.30 ± 5.12	<0.0001	36.69 ± 7.20	33.28 ± 6.87	<0.0001	<0.0001	0.74
BMI Classes			0.0003 ^α^			0.002 ^α^	0.87 ^¥^	0.37 ^¥^
Normal Weight (<25.0)	0 (0.00)	0 (0.00)		0 (0.00)	0 (0.00)			
Overweight (25.0–30.0)	11 (14.67)	24 (32.00)		5 (13.51)	15 (40.54)			
Obese (>30.0)	64 (85.33)	51 (68.00)		32 (86.49)	22 (59.46)			
Waist (cm)	107.54 ± 12.60	99.31 ± 12.61	<0.0001	119.47 ± 12.84	109.39 ± 12.87	<0.0001	<0.0001	0.0002
Systolic pressure (mmHg)	128.43 ± 12.47	122.39 ± 9.62	<0.0001	133.46 ± 11.09	124.73 ± 7.65	<0.0001	0.01	0.28
Diastolic pressure (mmHg)	80.34 ± 10.00	75.12 ± 7.51	<0.0001	85.81 ± 8.62	78.98 ± 5.72	<0.0001	0.005	0.01
Smoke (Yes) (%)	11 (14.67)	--	--	14 (37.84)	--	--	0.006	--
Glycemia (mg/dL)	92.77 ± 8.76	87.31 ± 9.50	<0.0001	96.35 ± 11.33	89.89 ± 10.26	<0.0001	0.13	0.18
Insulin (µU/mL)	15.66 ± 9.38	9.35 ± 4.69	<0.0001	19.58 ± 11.88	10.72 ± 5.09	<0.0001	0.05	0.16
HOMA	3.60 ± 2.18	2.04 ± 1.06	<0.0001	4.70 ± 3.06	2.41 ± 1.24	<0.0001	0.03	0.11
HbA1c (%)	5.42 ± 0.35	5.22 ± 0.35	<0.0001	5.50 ± 0.42	5.28 ± 0.35	<0.0001	0.45	0.67
Triglycerides (mg/dL)	96.83 ± 40.55	76.12 ± 28.02	<0.0001	140.35 ± 94.23	101.05 ± 42.83	0.0008	0.02	0.001
Total cholesterol	193.28 ± 40.67	166.02 ± 37.01	<0.0001	197.53 ± 55.97	169.41 ± 41.75	<0.0001	0.42	0.28
HDL (mg/dL)	57.02 ± 14.63	49.23 ± 11.30	<0.0001	45.01 ± 1.09	41.87 ± 9.38	0.02	<0.0001	0.0001
LDL (mg/dL)	127.61 ± 31.92	106.96 ± 29.97	<0.0001	139.89 ± 35.69	117.32 ± 26.54	0.0002	0.08	0.05
Creatininemia (mg/dL)	0.73 ± 0.10	0.73 ± 0.10	0.99	0.90 ± 0.13	1.11 ± 1.02	0.02	<0.0001	<0.0001
FM (Kg)	39.76 ± 11.17	33.51 ± 10.24	<0.0001	39.29 ± 15.30	31.59 ± 13.61	<0.0001	0.45	0.30
FFM (kg)	51.85 ± 5.50	50.33 ± 4.96	0.0004	72.15 ± 9.71	69.00 ± 10.59	0.0008	<0.0001	<0.0001

* Mean and Standard Deviation for continuous variables and frequency and percentage (%) for categorical variables. ^^^ Wilcoxon matched-pairs signed-rank test; ^†^ Wilcoxon rank-sum test (Mann–Whitney) among the parameters recorded before the diet by gender; ^ѱ^ Wilcoxon rank-sum test (Mann–Whitney) among the parameters recorded after the diet by gender; ^¥^ Chi-Square test; ^α^ McNemar–Bowker test. Abbreviations: BMI, Body Mass Index; IPAQ, International Physical Activity Questionnaires; HOMA, Homeostatic Model Assessment; HbA1c, Hemoglobin A1c; HDL, High-Density Lipoprotein; LDL, Low-Density Lipoprotein; FM, Fat Mass; FFM, Free Fat Mass.

**Table 2:** Liver parameters of patients undergoing the ketogenic diet, stratified by gender.

Parameters *	Gender			Gender			*p* ^†^	*p* ^ѱ^
	F (*n* = 75)			M (*n* = 37)				
	Before	After	*p* ^^^	Before	After	*p* ^^^		
Uricaemia (mg/dL)	4.71 ± 1.02	5.34 ± 1.13	0.0004	6.32 ± 1.10	6.88 ± 1.47	0.19	<0.0001	<0.0001
AST (mg/dL)	19.00 ± 8.86	18.04 ± 5.61	0.91	26.97 ± 12.17	22.43 ± 7.85	0.006	<0.0001	0.005
ALT (mg/dL)	23.43 ± 18.11	19.32 ± 9.16	0.002	43.57 ± 28.77	32.84 ± 19.56	0.003	<0.0001	<0.0001
γGT (mg/dL)	20.52 ± 13.04	14.13 ± 6.14	<0.0001	33.76 ± 19.53	20.30 ± 10.81	<0.0001	<0.0001	0.0004
CRP (mg/dL)	1.14 ± 6.18	1.08 ± 5.81	0.42	0.35 ± 0.36	0.28 ± 0.26	0.17	0.27	0.02
Ferritin (mg/dL)	68.80 ± 53.42	86.52 ± 67.67	<0.0001	306.77 ± 221.55	298.09 ± 184.86	0.99	<0.0001	<0.0001
CAP (dB/m)	270.87 ± 55.19	224.97 ± 55.79	<0.0001	308.70 ± 57.57	246.16 ± 66.27	<0.0001	0.001	0.05
E	5.69 ± 2.97	5.34 ± 2.14	0.24	6.73 ± 3.51	5.36 ± 1.93	0.01	0.02	0.82

* Mean and Standard Deviation for continuous variables and frequency and percentage (%) for categorical variables. ^^^ Wilcoxon matched-pairs signed-rank test; ^†^ Wilcoxon rank-sum test (Mann–Whitney) among the parameters recorded before the diet by gender; ^ѱ^ Wilcoxon rank-sum test (Mann–Whitney) among the parameters recorded after the diet by gender; Abbreviations: AST, Aspartate Aminotransferase; ALT, Alanine Transaminase; γGT, Gamma-Glutamyl Transferase; CRP, C-Reactive Protein; CAP, Controlled Attenuation Parameter; E, Liver stiffness.

**Table 3:** Linear regression analysis of CAP value on gender insert single in a model adjusted ^.

Parameters	CAP			
	β	se (β)	*p*	95% C.I.
Gender (M)	23.96	10.06	0.019	4.00 to 43.91

Abbreviations: CAP, Controlled Attenuation Parameter; β, Coefficient; se (β), Standard Error of Coefficient β; 95% C.I., Confidential Interval at 95%. ^ Models adjusted for Age, Smoking, BMI, and HOMA.

## Data Availability

The original contributions presented in this study are included in the article. Further inquiries can be directed to the corresponding author.
